# The effect of perioperative intravenously administered iron isomaltoside 1000 (Monofer®) on transfusion requirements for patients undergoing complex valvular heart surgery: study protocol for a randomized controlled trial

**DOI:** 10.1186/s13063-018-2545-3

**Published:** 2018-07-04

**Authors:** Seung Hyun Lee, Jae-Kwang Shim, Sarah Soh, Jong Wook Song, Byung Chul Chang, Sak Lee, Young-Lan Kwak

**Affiliations:** 1 Department of Thoracic and Cardiovascular Surgery, Severance Cardiovascular Hospital, Yonsei University Health System, College of Medicine, 50-1, Yonsei-ro, Seodaemun-gu, Seoul, 03722 Republic of Korea; 2 Department of Anesthesiology and Pain Medicine, Anesthesia and Pain Research Institute, Yonsei University Health System, Yonsei, Republic of Korea

**Keywords:** Complex valvular heart surgery, Iron isomaltoside 1000, Anemia, Transfusion

## Abstract

**Background:**

Anemia is a frequent complication after cardiac surgery especially following reoperation due to previous prosthetic valve failure or multiple valve surgery (including combined coronary artery bypass grafting). This trial explores whether intravenously administered iron isomaltoside 1000 (Monofer®) results in better clinical outcomes in patients undergoing complex heart valve surgery who are expected to receive transfusion.

**Methods/design:**

In this prospective, single-center, double-blinded, randomized controlled trial, 214 patients undergoing reoperation or multiple valve surgery are randomly allocated to either the iron isomaltoside 1000 (IVFe) or the control group from August 2016 to August 2018. The IVFe group receives iron isomaltoside 1000 mg (maximum dose 20 mg/kg) intravenously 3 days before and after the surgery. The control group receives an equivalent volume of normal saline. The primary endpoint is transfusion requirement (more than 1 unit of packed erythrocytes) for postoperative care until discharge and secondary endpoint are major complications, such as delayed ventilator therapy, acute kidney injury, and mortality. Reticulocyte count, plasma hepcidin, iron profiles (serum iron, serum ferritin, total iron-binding capacity, transferrin, transferrin saturation), coagulation profiles, urinary analysis, and chemical profiles are measured for three preoperative baseline-data days and just before surgery, except for hepcidin. After surgery, daily routine basic laboratory tests are measured just before discharge and reticulocyte count, iron profiles, and hepcidin are repeatedly checked for three postoperative days.

**Discussions:**

From our study, we can clarify the following points: the first is the perioperative IVFe effect on the demand for transfusion, and clinical outcomes in reoperation or complex valve surgery and the second is the role of hepcidin in the effect of IVFe on the hemoglobin level increase.

**Trial registration:**

ClinicalTrials.gov, Identifier: NCT02862665. Registered on August 2016.

**Electronic supplementary material:**

The online version of this article (10.1186/s13063-018-2545-3) contains supplementary material, which is available to authorized users.

## Background

Anemia is a common postoperative disorder in cardiac surgery patients after cardiopulmonary bypass (CPB) [1, 2]. Allogeneic blood transfusions (ABT) with red blood cells, which should be avoided whenever possible, is associated with complications [3] because ABT carries increased risks for transmission of infectious diseases and has been clearly demonstrated to be associated with adverse outcomes related to postoperative acute kidney injury (AKI), neurological complications, atrial fibrillation (AF), acute lung injury, and increased mortality [4–6]. So, many strategies have been developed to minimize the need for ABT, but transfusion requirements still remain high [3].

In patients with cardiovascular disease, iron deficiency is common either due to depletion of whole-body iron stores (absolute iron deficiency) or because of restricted availability of iron for erythrogenesis (functional iron deficiency). Thus, iron therapy is commonly used to reinforce the iron reserve to prevent anemia after bleeding; however, the effect of iron replacement after cardiovascular surgery is not clear. Iron plays an essential role in erythropoiesis and hemoglobin (Hb) synthesis [7]. The route of iron administration and the type of iron preparation vary widely. The effectiveness of iron supplement administration per os is often limited by its gastrointestinal side effects (10–40%) and low absorption rate (10–15%) [8, 9]. Therefore, some authors have suggested a role for intravenously administered (IV) iron in correcting anemia after orthopedic and oncological surgery, and in renal and obstetric patients [10, 11], but this route still remains controversial in cardiac surgery. In addition, the iron-regulatory protein, hepcidin, is responsible for controlling dietary iron absorption and body iron distribution [12]. Hepcidin acts by binding to the iron transport protein ferroportin, blocking iron absorption from the intestine and iron release from macrophages, leading to reduced iron delivery to erythroid precursors [13]. This hepcidin’s role under IV iron replacement also remains unclear in patients undergoing cardiovascular surgery.

Therefore, in this study, we tried to investigate the effect of the perioperative administration of IV iron isomaltoside 1000 (Monofer®; Pharmacosmos, Holbaek, Denmark) in patients undergoing re-do or a complicated valve replacement such as coronary bypass grafting (CABG) combined, or cases of multiple valve replacement and arrhythmia surgery combined. We also tried to assess the role of hepcidin as a biomarker, as well as any association of hematological parameters with outcome.

## Methods/design

### Study design

This prospective, double-blind, placebo-controlled, randomized, comparative, single-center trial was planned in Severance Cardiovascular Hospital, Seoul, Korea from August 2016 to August 2018 in patients undergoing re-do (re-do, second re-do, etc.) – or complicated valve replacement (double valve – aortic, mitral, with or without tricuspid surgery, CABG combined, arrhythmia surgery combined) cases. The trial protocol and related documents were approved by the competent authorities and Institutional Review Board (IRB) of the University of Yonsei (approval number: 4-2016-0502). The trial was conducted in accordance with Good Clinical Practice and the Declaration of Helsinki. Informed consent was obtained in writing prior to any trial-related activities. This trial was registered at ClinicalTrials (ClinicalTrials.gov, Identifier: NCT02862665) in August, 2016.

Patients will be randomly allocated to either the IV iron isomaltoside 1000 (IVFe) or the control (normal saline) group in a 1:1 ratio by means of computer-generated random numbers. IVFe administration is scheduled at 3 days before and after surgery. The patients will be checked by laboratory testing five times perioperatively at: (1) the preoperative baseline visit: reticulocyte count, Hb, plasma hepcidin, iron profiles (serum iron, serum ferritin, total iron-binding capacity, transferrin, transferrin saturation), coagulation profiles (PT, PTT), routine urinary analysis, and chemical profiles (aspartate aminotransferase (AST)/alanine aminotransaminase (ALT), serum creatinine, electrolytes, C-reactive protein (CRP), creatine kinase (CK), estimated glomerular filtration rate (GFR)), (2) start of therapy (first injection of IVFe 3 days before surgery): repeat laboratory test except for plasma hepcidin, (3) postoperative day 3 (just before the second injection of IVFe): repeat laboratory test except for urinary analysis, (4) postoperative day 10 or discharge day: repeat laboratory test except for plasma hepcidin and urinary analysis, and (5) postoperative week 4: repeat laboratory test except for plasma hepcidin and urinary analysis (Table 1).

**Table 1:** Perioperative laboratory data sheet

	Lowest Hb/HCt	reticulocyte count	Highest PT (sec/INR)	Highest PTT	Highest GOT/GPT	Highest Cr	U/A RBC(±)	Serum iron	Transferrin sat (%)	Ferritin (ng/ml)	TIBC^a^	Hepcidin
Before surgery 2–3 days	/		/		/		(±)					
Just before surgery	/		/		/		(±)					
POD0	/		/		/		(±)					
POD1	/		/		/		(±)					
POD2	/		/		/							
POD3	/		/		/							
POD4	/		/		/							
POD5	/		/		/							
POD6	/		/		/							
POD7	/		/		/							
POD10 or discharge day	/		/		/							
Follow-up OPD (20)	/		/		/							

*U/A RBC* Urinary analysis red blood cells, *GOT* Glutamic Oxalacetate Transaminase, *GPT* Glutamic Pyruvate Transaminase

*Cr* creatinine, *Hb* hemoglobin, *HCt* hematocrit, *INR* International Normalized Ratio*, OPD* outpatient department, *OT, POD* postoperative day*, PT* prothrombin time, *PTT* partial thromboplastin time, *TIBC* total iron-binding capacity*, U/A RBC* Urinary analysis red blood cells.

The IVFe group received iron isomaltoside 1000 as a single-dose infusion of 1000 mg over 30 min with a maximum single dose of 20 mg/kg. The placebo group received saline (0.9% Sodium Chloride Injection; JW Pharmaceutical, Seoul, Korea) as a single-dose infusion of 100 ml over 30 min.

### Participants and eligibility criteria

**Fig. 1:**
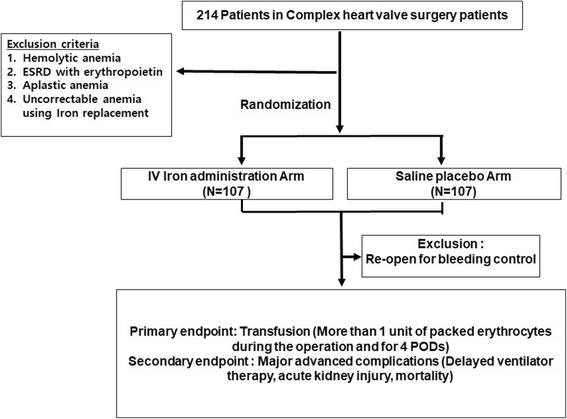
Schematic of study design

Basically, all patients aged 19 years of age or older undergoing re-do (re-do, second re-do, etc.) – or a complicated valve replacement (double valve – aortic, mitral, with or without tricuspid surgery, CABG combined, arrhythmia surgery combined cases) are included. Eligibility will be determined by two cardiac surgeons and two cardiac anesthesiologists on the basis of the preoperative evaluation results. After completing the screening, participants will be guided through the informed consent process and written informed consent will be obtained from all participants before the initiation of any preoperative iron treatment. After consent forms are signed, participants will be randomly allocated to one of the two groups in a 1:1 ratio (Fig. 1).

### Exclusion criteria

Exclusion criteria are as followings: (1) patients who have critical anemia that cannot be corrected by only iron replacement therapy (Hb < 9 g/dl for women and Hb < 10 g/dl for men) such as aplastic anemia, (2) patients who have end-stage renal disease (ESRD) treated with erythropoietin or any concomitant medication for erythropoiesis, (3) patients with previous paravalvular leakage, because any ensuing hemolytic anemia cannot be corrected by iron replacement, (4) patients with a hyperallergic reaction to iron agents, and aplastic anemia, and (5) patients who have been treated in intensive care unit for severe cardiac dysfunction.

### Randomization

Patients are randomly allocated to either the IV iron isomaltoside 1000 (IVFe) or the control (normal saline) group in a 1:1 ratio by means of computer-generated random numbers. Random numbers will be generated by an independent statistics professional using SPSS version 19.0 for Windows (release 19.0 K; SPSS Inc. Armonk, NY, USA). Sealed assignment envelopes will be used for allocation concealment.

### Withdrawal and dropout

All participants will have the right to withdraw from the study at any time. Participation will be terminated if the individual wants to stop and withdraws consent. The trial will be stopped if the investigator believes that there are unacceptable risks to participants. If any patients show typical complications of IVFe, such as nausea, vomiting, hypotension, gastric discomfort, urticaria, and acute renal failure, participation will be terminated immediately. The patients who have Hb concentrations higher than 8 mg/dl postoperatively, yet require transfusion due to symptoms of acute anemia, such as shortness of breath, change in respiratory rate and pulse rate, mental function deterioration, and myocardial ischemia, are excluded from the study. Patients with postoperative paravalvular leakage should be removed to avoid confusion with the possibly similar effects on red blood cells as hemolytic anemia. Postoperative gastrointestinal bleeding is excluded. We also excluded patients who underwent re-exploration for postoperative bleeding (postoperative bleeding is defined as total drainage volume < 1000 cm^3^ during the 4 h after arrival in the intensive care unit (ICU)).

### Intervention

IVFe administration is scheduled twice, at 3 days before and after surgery, regardless of Hb level. The IVFe group receives iron isomaltoside 1000 as a single-dose infusion of 1000 mg over 30 min with a maximum single dose of 20 mg/kg. The placebo group receives saline (0.9% Sodium Chloride Injection; JW Pharmaceutical, Seoul, Korea) as a single-dose infusion of 100 ml over 30 min.

### Study objective and outcomes (primary and secondary outcomes)

The primary objective of the trial is to demonstrate that IVFe is superior compared to placebo in leading to less decrease in the Hb level and reducing the need of transfusion in patients undergoing complex cardiac surgery, which is a highly possible scenario for receiving a transfusion. The secondary objectives are to compare the effects of IVFe on the clinical (or surgical) outcomes (e.g., renal failure, respiratory failure, etc.) from reducing the need for blood transfusion and the stability of iron-related parameters. The final objective is to assess the role of the iron-regulatory protein, hepcidin, as a biomarker, as well as any association of hematological parameters with outcome. Thus, we decided the endpoints as followings: the primary endpoint is the comparison of perioperative transfusion requirements in terms of overall incidence and mean amounts of units of packed erythrocytes transfused per patient during and after the operation until discharge, and the secondary endpoint is the occurrence of postoperative major advanced complications (delayed ventilator therapy, AKI and all-cause mortality). A Hb concentration of less than 7 mg/dl during CPB, and less than 8 mg/dl after CPB and postoperatively, were used as transfusion thresholds. Intraoperatively, we measured the Hb concentration post induction and every 30 min during CPB, then at 10 min post CPB and post sternal closure, and postoperatively at the time of, and 8 h after, arrival at the ICU. After the day of surgery, we measured the Hb concentration in the morning if there was no excessive bleeding (over 1000 cm^3^) or symptoms of anemia. Any patients who have Hb concentrations higher than 8 mg/dl postoperatively, yet required transfusion due to symptoms of acute anemia, such as shortness of breath, change in respiratory rate and pulse rate, mental function deterioration, and myocardial ischemia, were excluded from the study. We also excluded patients who had undergone re-exploration for postoperative bleeding (postoperative bleeding was defined by total drainage volume (< 1000 cm^3^) during the 4 h after arrival in ICU).

Postoperative variables included the amount of bleeding measured by chest-tube drainage after surgery, fluid input, urine output, and overall amount of ABT. Hb concentrations, PT, AST/ALT, and creatinine (Cr) levels were measured preoperatively, post anesthetic induction, and postoperative day (POD) 0 (day of the surgery after arrival in the ICU), 1, 2, 3, 4, and 7. Reticulocyte count and iron profiles were measured preoperatively and post anesthetic induction and at POD 3, and POD 10, and the changes in reticulocyte count from the baseline values were calculated. Postoperative variables also included the incidence of perioperative transfusion volume, the incidence of AKI within 48 h after surgery, new-onset AF after surgery, duration of ventilator care, ICU stay and hospital stay, and in-hospital mortality. Multiple ABT is defined as more than 1 unit of packed erythrocytes during the operation and until discharge. AKI is defined as elevation of serum Cr of ≥ 0.3 mg/dl or 50–200% from baseline using the modified Risk, Injury, Failure, Loss, and ESRD classification [14]. Surgical mortality is defined as all deaths that occur during the hospital stay or after hospital discharge but within 30 days postoperatively.

### Data collection

We will collect the clinical data from the laboratory results of all enrolled patients during admission and at outpatient clinic follow-up.

### Sample size

Sample size estimation is based on the null and alternative hypotheses. In our study, the null hypothesis is that “IVFe has no significantly different effect on Hb level and transfusion need after complex cardiac surgery” and we want to prove that the null hypothesis is wrong for replacing the alternative hypothesis by the two-sided test method. Previous reports [15, 16] suggested a 35% reduction in transfusion rate using IVFe but we set a higher transfusion rate (40%) considering the complexity and severity of cardiac surgery. The sample size calculation was based on superiority analysis under the hypothesis that all data was normally distributed data. We set the significance level at 0.05, the power at 80%, and the effect size was 0.5. A two-sided test was used and consequentially 97 patients were need for each group; however, we set each group as 107 considering a 10% dropout rate (total 214).

### Statistical methods

Continuous variables were shown as means ± standard deviation (SD) and dichotomous variables are shown as numbers (percentages). Between-group comparisons of continuous variables were performed using the independent Student *t* test. Dichotomous variables were compared using the chi-square or Fisher’s exact tests, as appropriate. *P* values < 0.05 will be considered statistically significant.

## Discussion

ABT is a recognized risk factor for morbidity and mortality after cardiac surgery [6, 17]. Despite efforts to reduce ABT, it remains common in cardiac surgery. Several studies have been published about the effect of erythropoietic agents such as Recombinant Human Erythropoietin (rHuEPO) [18–20] and IV iron isomaltoside 1000 (Monofer®) [21, 22]. For rHuEPO, the theoretical basis for selecting the timing of rHuEPO administration was based on the assumption that a pre-emptive single dose of rHuEPO may mitigate the inflammatory response-induced blunting of erythropoiesis; however, this hypothesis remains speculative unless we also evaluate the representative inflammatory markers. Also, the potential risk of rHuEPO has always been considered a considerable burden including the possible complications of rHuEPO which include hypertension, headache, tachycardia, nausea, vomiting, hypercalcemia, diarrhea, and thromboembolic complications. Recently, therefore, direct IV iron replacement agents have received attention as alternatives to rHuEPO therapy for reducing transfusion need. In various surgical fields, the effect of IV iron replacement therapy on clinical outcomes by reducing ABT have been analyzed and they showed favorable results compared with simple iron-replacement therapy per os. In the study by Muñoz et al., ABT, postoperative nosocomial infection, 30-day mortality, and length of hospital stay (LHS) from 2547 patients undergoing elective lower-limb arthroplasty (*n* = 1186) or hip fracture repair (*n* = 1361) were compared between patients who received either very-short-term perioperative IV iron (200–600 mg; *n* = 1538), with or without rHuEPO (40,000 IU), or standard treatment (*n* = 1009). They suggested that very-short-term perioperative administration of IV iron, with or without rHuEPO, in major lower-limb orthopedic procedures are associated with reduced ABT rates and LHS, without increasing postoperative morbidity or mortality [16].

Froessler et al. also analyzed whether perioperative IV iron reduced the need for ABT in abdominal surgery patients. Seventy-two patients with iron-deficiency anemia were assigned to the study which suggested that the administration of perioperative IV iron reduces the need for ABT, and is associated with a shorter LHS, enhanced restoration of iron stores, and a higher mean Hb concentration 4 weeks after surgery [15].

Focusing on the cardiac surgery itself, Johansson et al. explored whether IV iron isomaltoside 1000 (Monofer®) results in a better regeneration of Hb levels and prevented anemia compared to placebo in preoperative non-anemic patients undergoing cardiac surgery. They showed that a single, perioperative 1000-mg dose of IV iron isomaltoside 1000 significantly increased the Hb level and prevented anemia 4 weeks after surgery, with a short-term safety profile similar to placebo [21]. However, this trial was an exploratory trial with only a small sample size of 60 patients and relatively simple cardiac surgery such as CABG. So, the actual risk of bleeding is too low to perform transfusion compared with our protocol. Our protocol includes patients undergoing complicated valve surgery, such as re-operation from previous valve failure, combined with CABG, arrhythmia surgery, etc.; therefore, it is more easily reproducible to compare ABT rate between groups in the “real” surgical world. Also, our protocol contains an analysis of hepcidin effect in which it is unclear how hepcidin affects the IVFe as it works to increase the Hb and improve clinical outcomes.

### Study status

This study is an ongoing trial and patient recruitment remains incomplete at the time of submission (Additional file 1).

## Additional file

Additional file 1: SPIRIT checklist. (DOCX 60 kb)

## Abbreviations

ABT: Allogeneic blood transfusions
AF: Atrial fibrillation
AKI: Acute kidney injury
CABG: Coronary bypass grafting
CPB: Cardiopulmonary bypass
Cr: creatinine
ESRD: End-stage renal disease
Hb: Hemoglobin
IRB: Institutional Review Board
IV: Intravenously administered
IVFe: IV iron isomaltoside 1000
LHS: Length of hospital stay
POD: Postoperative days
rHuEPO: Recombinant Human Erythropoietin
